# Pathology of Synchisis

**Published:** 1847-11

**Authors:** 


*Pathology of Synchisis.*—The Comptes Rendus contain an abstract of an interesting paper read before the Academy of Sciences, at the séance of July 19th, by M. Bouisson, on that affection of the vitreous body known as synchisis.

It has long been desired to make out the real nature of this abnormal condition, and various hypotheses have been brought forward.

M. Bouisson had entertained the idea that the moveable and sparkling particles observed deep-seated in the eye, in persons affected with this malady, were not caused by loose floating particles of the hyaloid membrane, but constituted of free crystalline morsels in the thickness of the vitreous body, the membrane of which was destroyed.

Upon devoting himself to some researches on the composition of the vitreous humour, he recognised the existence of a fatty matter, in such a state of minute division that the transparency of the humour was not affected by it. After having filtered the vitreous humour of the eye of an ox, he evaporated it in a porcelain capsule, and treated the residue with sulphuric ether. The resulting matter was then collected in a watch-glass, perfectly clean, and evaporated. During the process of evaporation, the ether deposited a fatty matter in a crystalline form. The same experiment upon a larger quantity of vitreous humour, obtained from several oxen's eyes, gave the same result, but more clearly. M. Bouisson has also obtained this fatty matter, by treating the vitreous humour of the human eye in the same manner.

If these results be taken in connexion with the observations which demonstrate that crystals of cholesterine have been found in the posterior chamber of eyes which have for a long time been struck with blindness, one will naturally be led to believe, that in the normal condition a certain quantity of fatty matter is contained in the vitreous humour, which may be separated in a crystalline form by some peculiar pathological influence, and may acquire, in this form, that apparent mobility at the bottom of the eye which arrests our attention.

From these facts and considerations the nature of synchisis may be determined, for we have grounds to believe that this singular malady is due to the accidental deposition of the fatty matter of the vitreous humour in a crystalline form.

Synchisis, or synchesis, is described as a dissolved state of the vitreous body, in which the hyaloid membrane is either atrophied or destroyed; hence it is that the vitreous humour appears diffluent in form, and wanting in its natural consistence. Although this condition may result from inflammatory action, still it is more frequently met with in the eyes of the aged, as a natural decay or degeneration, and often in company with cataract—another instance of the failure of vital energy. Now, if the above observations of M. Buisson be confirmed, synchisis will afford another instance of that very general abnormal condition—fatty degeneration of the tissues—which has lately been so much studied and illustrated.

Synchisis, as a disease of old age, will be brought to coincide with other lesions of the aged, also marked by the abnormal production of fat, from perverted nutrition—as, for instance, the degeneration and subsequent calcification of the arterial coats. And at the same time, synchisis, considered as a result of inflammation, will agree, as to it pathological characters, with the consequences of inflammatory action in other organs, and in that self same circumstance—viz., the unnatural development of fatty matter. In old age, we should ascribe the appearance of the crystals of fat in the vitreous humour, in an abnormal condition, to impaired nutrition, and consequent degeneration with atrophy; and, again, we should especially anticipate the occurrence of atrophy when the synchisis is accompanied by cataract, whereby the function of the vitreous body is destroyed.—*London Lancet.*

